# How to Cure Rhus Poisoning

**Published:** 1893-08

**Authors:** R. F. Rabe

**Affiliations:** Office of Rabe & Keller, Counselors-at-Law, 243 Broadway, New York


### HOW TO CURE RHUS POISONING.

Office of Rabe & Keller, Counselors-at-Law, 243 Broadway, New York, June 24th, 1893.

To the Editor of Homœopathic Physician:

The inclosed clipping is taken from the *Literary Digest* of June 24th. Comment unnecessary.

Poison Ivy—How to Cure the Poisoning.—Procure from the drug or other stores where they are sold *a small box of little sugar pills labeled* "Rhus-tox." A "hair of the dog that bit you" will cure you. Take six of the little pills at one dose, four doses the first day—morning, noon, evening, and bed-time. The next day the itching will be mollified a degree. The second and third day, take three doses of six pills each dose. You will, by this time, be so free from irritation that you may carelessly take a few pills until nature heals up the sores. As soon as the healing begins be very chary of taking many of the pills, as they will, in excess of requirement, produce an intolerable, though harmless, itching over the whole body.—*H. M., in Scientific American, New York, June* 17th.

Why is Homœopathy not acknowledged in this case?

Respectfully yours,

R. F. Rabe, Jr.

